# Distinct effects of short-term reconstructed topsoil on soya bean and corn rhizosphere bacterial abundance and communities in Chinese Mollisol

**DOI:** 10.1098/rsos.181054

**Published:** 2019-01-30

**Authors:** Zhenhua Yu, Jian Jin, Yansheng Li, Yue Yang, Yue Zhao, Changkai Liu, Xiaobing Liu

**Affiliations:** Key Laboratory of Mollisols Agroecology, Northeast Institute of Geography and Agroecology, Chinese Academy of Sciences, Harbin 150081, People's Republic of China

**Keywords:** soil erosion, topsoil thickness, Mollisol, bacterial community, 16S rRNA gene sequencing

## Abstract

Eroded black soils (classified as Mollisols) lead to a thinner topsoil layer, reduced organic carbon storage and declined crop productivity. Understanding the changes in soil microbial communities owing to soil erosion is of vital importance as soil microbial communities are sensitive indicators of soil condition and are essential in soil nutrient cycling. This study used the reconstructed facility with 10, 20 and 30 cm topsoil thickness under no-till soya bean–corn rotation in black soil region of Northeast China. Illumina MiSeq sequencing targeting 16S rRNA, *q*PCR and soil respiration measurement were performed to assess the changes in soya bean and corn rhizosphere bacterial communities, as well as their abundance and activities due to the topsoil thickness. The results showed that soil bacterial communities from both soya bean and corn were more sensitive to topsoil removal than to soil biogeochemical characteristics. Topsoil depths significantly influenced both soya bean and corn bacterial communities, while they only significantly influenced the bacterial abundance and respiration in corn. We also found that the topsoil depths significantly induced the changes in phyla and genera from both soya bean and corn rhizosphere bacterial community, which aid further understandings on how topsoil layer influences the global nutrient cycling of Mollisols by influencing the change in microbial communities.

## 1. Introduction

The degradation of soil by erosion is a threat worldwide due to its negative effects on the environment and crop productivity [1]. Erosion causes the decrease in the topsoil thickness [2]. Cotching *et al*. [3] found that the thickness and organic matter content of topsoil had a positive correlation with soil productivity. Topsoil thickness is of vital importance in maintaining the soil quality and productivity [4]. The northeastern region of China, where the fertile and productive Mollisols are primarily distributed, is the main grain production base in China. In 2016, the regional corn and soya bean yields were 51% and 37%, respectively, of the national total (National Bureau of Statistics of China, 2016). Although the history of agricultural cultivation in this region dates back only 100 years, this region suffers from serious soil degradation problem, especially soil erosion, which decreases the thickness of humus horizon layer from 60–100 cm to 20–40 cm, as well as the decline of soil nutrient level or soil biophysico-chemical properties, such as soil organic matter content, microbial activities and soil water storage capacity [3–5]. These changes directly influence the growth of plant root system and rhizosphere environment, reduce soil productivity and increase the cost of chemical input as a result, which definitely induce adverse effects in the sustainable development of agriculture food security in this region.

Soil microbial communities are sensitive indicators of soil condition [6] and are essential in soil carbon/nitrogen (C/N) cycling processes and nutrient transformation. Any alteration in the composition and activity of soil microbial communities is likely to influence plant nutrient availability [7], soil C-storage [8] and ecosystem productivity and functioning [9]. Therefore, understanding the changes in soil microbial communities owing to soil erosion is of vital importance to soil microenvironment, and thus root growth and development. In the hilly red soil region of southern China, Li *et al*. [10] investigated the microbial responses in relation to soil physico-chemical property changes induced by erosion. In black soil region of Northeast China, extensive studies have been carried out in characterizing simulated soil erosion on soil productivity [11], and the effectiveness of soil amendments for restoring the productivity of eroded soils [12]. These studies proposed the declined productivities in eroded Mollisol varied among crop types. However, little information is available on the response of soil microbial community or activity in soya bean and corn to soil erosion in this region.

Therefore, this study aimed to clarify the responses of soil microbial community to topsoil thickness in soya bean and corn rotation system. We hypothesized that (1) topsoil with different thickness will change the microbial community, (2) thicker topsoil depth will enhance the microbial activities and increase the microbial abundance, (3) the responses of soil microbial communities to topsoil thickness will be different between soya bean and corn. To test these hypotheses, we used the reconstructed topsoil facility with three topsoil thickness under no-till soya bean–corn rotation system in Heilongjiang Province, a typical region of Mollisol in Northeast China. Illumina MiSeq sequencing targeting 16S rRNA, real-time PCR (*q*PCR) and soil respiration measurement were performed to characterize the bacterial communities, as well as their abundance and activities during the growing season of soya bean and corn.

## 2. Material and methods

### 2.1. Experimental design

The research site is located in Guangrong Village, Hailun City, Heilongjiang Province of Northeast China (47°26′ N, 126°38′ E). The site is within the north temperate zone and continental monsoon area, which is cold and arid in winter and hot and rainy in summer. The annual average temperature is 1.5°C, ranging from 32°C in summer to −37°C in winter. The annual mean rainfall for the last decade was 548 mm, and the average temperature and rainfall during the growing season (from 1 May to 30 September) were 18.1°C and 474 mm, respectively. The soil is a typical Chinese Mollisol on a 5° slope with 30 cm thick A horizon.

The simulation experiment was set up in April 2015. Depths of simulated topsoil were 10, 20 and 30 cm deep. The selection of these soil depths was based on the survey that nearly 40% topsoil thickness in the farmland of black soil region is less than 30 cm deep. Topsoils with 30 cm depths in the designed area were all removed by bucket grader and then mixed well. Then certain soil mass was refilled back to each plot according to designed topsoil depth. Soil mass for each topsoil depth reconstruction was calculated on the soil bulk density, the depths of topsoil and the area of the reconstructed plot. Each plot was 10.4 m in length and 7.5 m in width. Every soil depth treatment was replicated thrice. After the topsoil depth was reconstructed, the surface soil was flattened, and the soil was naturally deposited for 1 year with soya bean planted to cover the soil. All soya bean residues were threshed and returned to cover the soil after harvest. Field experiment was started in the growing season of 2016. Popular cultivars of soya bean (Dongsheng 1) and corn (Xinken 5) in the region were planted. The crop sequences of soya bean–corn and corn–soya bean rotations were implemented in two independent blocks in the field. Basal nutrients were applied at the following rates (kg ha^−1^): 150 (NH_4_)_2_HPO_4_, 50 urea and 50 K_2_SO_4_ for soya bean, and 150 (NH_4_)_2_HPO_4_, 75 urea and 50 K_2_SO_4_, with 75 urea as the top dressing for corn.

### 2.2. Soil sampling and characteristics

In the year 2017, at the R6 stage of soya bean and R3 stage of corn, several shoots were cut off, and the roots were carefully separated from soils. Only the soil adhering to the roots was considered as rhizosphere soil [13] and the rhizosphere soils were collected by shaking the roots. A part of composite soil samples was placed into autoclaved microcentrifuge tubes (2 ml) immediately. The tubes were stored at −80°C until use. Another part of the soil was kept at 4°C for further physico-chemical property analysis and microbial biomass C determination.

### 2.3. Soil physico-chemical properties and microbial biomass C determination

Soil moisture was measured gravimetrically. A proportion of fresh soil samples was obtained for the measurements of the microbial biomass C and N (microbial biomass carbon/N) [14] and ammonium ($NH_{4}^{+}-N$) and nitrate ($NO_{3}^{-}-N$) concentrations (SKALAR, San^++^, Netherlands). The rest of the soil samples were air dried for determining Olsen phosphorus (P) [15]. Soil pH was measured using a pH meter after shaking the soil water (1:5 v/v in H_2_O) suspension for 30 min. Available potassium (AK) and other additional soil chemical properties were determined using methods described by Lu [16].

### 2.4. Soil incubation and respiration measurements

Sieved soil (20 g) was placed into PVC cores (4.5 cm height, 2 cm diameter) with nylon mesh bottoms. Each PVC core was placed into a 0.25 l wide-mouth mason jar together with a vial containing 10 ml of water to maintain the humidity inside the jar. The soil water content was maintained at 80% field water capacity by weighing and watering. A 15 ml plastic vial containing 10 ml 1 M NaOH was placed in each jar as a base trap to capture evolved CO_2_. All mason jars were placed in an incubator under dark conditions at a constant temperature of 25°C. Soil respiration was estimated by measuring the amount of CO_2_ absorbed in the NaOH trap. Titrations were performed after 24 h incubation. The CO_2_ trapped in the NaOH solution was precipitated with 0.5 M SrCl_2_ solution. HCl (0.1 M) was used to neutralize the excess NaOH using phenolphthalein as an indicator [17].

### 2.5. Soil DNA extraction

Soil DNA of each treatment (three replicates) was extracted from 0.5 g of frozen soils with a Fast DNA SPIN Kit for Soil (Qbiogene Inc., Carlsbad, CA, USA) according to the manufacturer's instructions. The extracted DNA was diluted in 20 µl TE (10 mM Tris-HCl, 1 mM EDTA, pH 8.0) buffer and stored at −20°C until use.

### 2.6. *q*PCR

*q*PCR was performed on a LightCycler^®^480 System (Roche) using primers 338F (5′-CCT ACG GGA GGC AGC AG-3′) and 518R (5′-ATT ACC GCG GCT GCT GG-3′) [18] by targeting the V3–V5 region of the 16S rRNA gene [19]. Each 20 µl reaction mixture contained 10 µl of SYBR Premix EX Taq TM (Takara, Dalian, China), 0.4 µl each of 10 µM forward and reverse primers, 2 µl of 100-fold diluted template DNA and 7.2 µl of sterilized Milli-Q water. The PCR was performed in triplicate, and the conditions were as follows: 95°C for 30 s, followed by 30 cycles of 95°C for 5 s, 60°C for 30 s and 50°C for 30 s. A melting curve analysis and agarose gel electrophoresis of the PCR products were conducted to confirm that the fluorescence signal was originated from specific PCR products and not from primer-dimers or other artefacts. The copy number of bacterial 16S rRNA genes was calculated using a regression equation to convert the cycle threshold (Ct) value to a known number of copies in the standards [20].

### 2.7. Illumina MiSeq sequencing

DNA extracted from each sample was used as a template for PCR amplification using the primers 515F/907R [21] by targeting the V4–V5 region of 16S rRNA gene [22]. The primers were modified with a unique 6-nt barcode at the 5′-end. An aliquot of 10 ng of purified DNA template from each sample was amplified in a 25 ml reaction system under the following conditions: initial denaturation at 95°C for 5 min, followed by 30 cycles consisting of denaturation at 95°C for 1 min, annealing at 63°C for 1 min and extension at 72°C for 1 min, with a final extension at 72°C for 5 min. Each sample was amplified in triplicate, and the PCR products were pooled and purified using an agarose gel DNA purification kit (TaKaRa, Dalian, China). An equimolar amount of the PCR products was combined into one pooled sample and submitted to Majorbio Bio-pharm Technology Co. Ltd (Shanghai, China) for Illumina paired-end sequencing (2 × 250) using the Illumina MiSeq platform.

### 2.8. Processing the DNA-sequence and diversity indices

After sequencing was completed, all sequence reads were quality checked using the quantitative insights into microbial ecology (QIIME) pipeline Version 1.8.0 (http://qiime.org/tutorials/tutorial.html) [23]. Briefly, any ambiguous reads or low-quality sequences shorter than 200 bp in length and with an average quality score of less than 20 were excluded from further analysis. Bacterial sequences with the same barcode were assigned to the same sample, and then the barcode and primer sequences were removed. Sequences with similarities of greater than 97% were clustered into one operational taxonomic unit (OTU). Coverage, Ace and Shannon indices were obtained using the Mothur program (http://www.mothur.org). Phylotypes were identified using Ribosomal Database Project (RDP) pyrosequencing pipeline (http://pyro.cme.msu.edu/). As the sequence number after quality check varied among samples, we randomly selected 21 971 and 25 688 sequences for soya bean and corn, respectively, based on their minimum reads before further analysis. Using the program R version 3.1.2 for Windows (R Development Core Team, 2010), principal coordinate analysis (PCoA) was processed to assess the patterns of similarity (Bray–Curtis similarity) in the composition of the microbial community between treatments. Mental test was applied to evaluate the correlations among microbial communities with environmental variables using PASSaGE (http://www.passagesoftware.net/). Differences in community structure were tested using ANOSIM [24] and ADONIS [25]. Analysis of the genus abundance with two-way ANOVA was performed using Genstat 12.0. The correlations between the genera and soil properties were examined using bivariate analysis by SPSS 16.0. DNA sequences have been deposited into the GenBank short-read archive SRP151783.

## 3. Results and discussion

### 3.1. Soil characteristics

Fundamental physico-chemical characteristics of the studied soils are summarized in table 1. $NO_{3}^{-}-N$ and total C content in the rhizosphere of soya bean were significantly affected by topsoil depths; the depth of 20 cm had the highest $NO_{3}^{-}-N$ content, and the total C significantly decreased with increasing topsoil depths. In the case of corn, remarkable differences in total potassium (TK), total carbon (TC) and pH were observed; TK and TC were the highest and pH was the lowest in topsoil with 10 cm depth. There was little or no effect on $NH_{4}^{+}-N$, available phosphorus, AK, total phosphorus, dissolved organic carbon (DOC) and total nitrogen contents.

**Table 1.** Physico-chemical characteristics of the soils from soya bean and corn rhizosphere.

	Topsoil depths	MBC (mg kg^−1^)	MBN (mg kg^−1^)	TP (mg kg^−1^)	TK (g kg^−1^)	AP (mg kg^−1^)	AK (mg kg^−1^)	DOC (mg kg^−1^)	DON (mg kg^−1^)	pH (1:2.5 v/v in CaCl_2_)	NH_4_^+^-N (mg kg^−1^)	NO_3_^−^-N (mg kg^−1^)	Total N (g kg^−1^)	Total C (g kg^−1^)
soya bean	10 cm	112 ± 9 a	17 ± 2 a	643 ± 17 a	23 ± 0.2 a	55 ± 3 a	141 ± 3 a	110 ± 11 a	30 ± 3 a	5.7 ± 0.2 a	6.5 ± 0.4 a	1.1 ± 0 b	1.8 ± 0.2 a	22.7 ± 0.8 a
	20 cm	80 ± 8 a	10 ± 2 a	613 ± 40 a	23 ± 0.3 a	54 ± 3 a	153 ± 5 a	81 ± 9 a	35 ± 2 a	6.2 ± 0.1 a	7.1 ± 0.2 a	4.9 ± 0.5 a	1.8 ± 0 a	21.2 ± 0.4 b
	30 cm	107 ± 10 a	20 ± 7 a	666 ± 18 a	24 ± 0.2 a	66 ± 6 a	160 ± 10 a	111 ± 7 a	28 ± 1 a	5.6 ± 0.2 a	6.3 ± 0.3 a	1.6 ± 0.1 ab	1.8 ± 0.1 a	20.9 ± 0.6 b
													
Corn	10 cm	112 ± 15 a	16 ± 3 a	672 ± 49 a	24 ± 0.1 a	44 ± 13 a	139 ± 8 a	67 ± 8 a	27 ± 7 a	5.5 ± 0 b	7.8 ± 1.6 a	4.0 ± 2.3 a	1.8 ± 0.1 a	24.0 ± 0.7 a
	20 cm	119 ± 15 a	17 ± 3 a	650 ± 20 a	23 ± 0.1 b	43 ± 8 a	157 ± 7 a	64 ± 6 a	34 ± 5 a	6.0 ± 0 a	8.2 ± 0.9 a	5.4 ± 0.9 a	1.78 ± 0 a	21.9 ± 0.9 ab
	30 cm	101 ± 21 a	12 ± 2 a	605 ± 13 a	23 ± 0.2 b	57 ± 5 a	156 ± 5 a	83 ± 4a	35 ± 8 a	6.1 ± 0.1 a	7.8 ± 0.9 a	5.9 ± 1.5 a	1.8 ± 0 a	21.5 ± 0.3 b

### 3.2. 16S rRNA gene sequence copy numbers and soil respiration

Partially supporting our second hypothesis, topsoil depths did not significantly influence the bacterial abundance and respiration in the rhizosphere of soya bean but significantly decreased the bacterial abundance and increased the respiration in corn, which indicated that corn was more susceptible to soil erosion than soya bean was (table 2). The higher sensitivity of corn to soil erosion was also confirmed by Sui *et al.* [12,26], who investigated the effect of topsoil removal on grain yield in the same zone and reported that crop yields declined with increased depth of topsoil removal and the yield reduction in corn was greater than in soya bean. Notably, the bacterial abundance in the rhizosphere of corn decreased, while the respiration in the rhizosphere of corn increased with the increase in topsoil depth. At the same topsoil depth, 16S rRNA gene sequence copy numbers in corn were significantly higher than those in soya bean and were higher than corn at the topsoil depth of 10 cm, while an opposite trend was noted for soil respiration. One explanation could be that the maize root weight density significantly increased with topsoil depths (Y Yang *et al.* 2019, unpublished), implying an intense competition between corn root growth and bacterial growth, thus only a few bacteria that had high competition ability and activities could survive under limited-nutrition conditions.

**Table 2.** Summary of number of OTUs, Chao and Shannon indices, 16S rRNA gene copies and respiration in the rhizosphere of soya bean and corn under topsoil depths of 10, 20 and 30 cm. The values are means of three replicates with the standard deviation in brackets. Values in the same column followed by the same letter are not significantly different among different topsoil depths.

	number of OTUs		Chao		Shannon		16S rRNA gene copies (×10^9^ copies g^−1^ dry soil)		respiration (ml kg^−1^ dry soil)	
topsoil depths (cm)	soya bean	corn	soya bean	corn	soya bean	corn	soya bean	corn	soya bean	corn
10	2274 (49) a	2485 (58) a	2997 (114) a	3217 (63) a	6.44 (0.04) a	6.55 (0.07) a	2.86 (1.28) a	4.07 (0.56) a	93.17 (11.61) a	46.24 (5.18) b
20	2239 (52) a	2494 (50) a	2981 (54) a	3237 (144) a	6.41 (0.04) a	6.54 (0.05) a	1.78 (0.25) a	3.52 (0.79) ab	80.26 (8.96) a	57.46 (17.14) ab
30	2321 (99) a	2489 (70) a	3147 (176) a	3232 (169) a	6.49 (0.05) a	6.56 (0.04) a	2.36 (1.00) a	2.49 (0.89) b	86.49 (3.93) a	81.73 (11.30) a

### 3.3. Bacterial community diversity analysis

The rarefaction coverage was greater than 0.97 for all the samples, revealing that the current number of sequence reads was sufficient to indicate bacterial diversity. Based on Mothur clustering, the number of OTUs was in the range of 2239–2321 and 2485–2494 for soya bean and corn, respectively. The bacterial diversity of soil samples was analysed by calculating the α-diversity indices (table 2). For the average number of OTU and Chao estimator, both soya bean and corn showed no significant differences among different topsoil depths. Shannon diversity indices were 6.41–6.49 and 6.54–6.56 for soya bean and corn, respectively. Similar to OTU numbers and Chao estimator, the Shannon diversity indices of soya bean and corn showed no significant differences among different topsoil depths. The relationship of OTU numbers and α-diversity with soil properties is shown in electronic supplementary material, table S1. Notably, DOC was observed to significantly correlate with OTU numbers, α-diversity, bacterial abundance and soil respiration as DOC was the main energy source for soil microorganisms and was considered to be an important indicator of soil quality [27].

**Figure 1.**
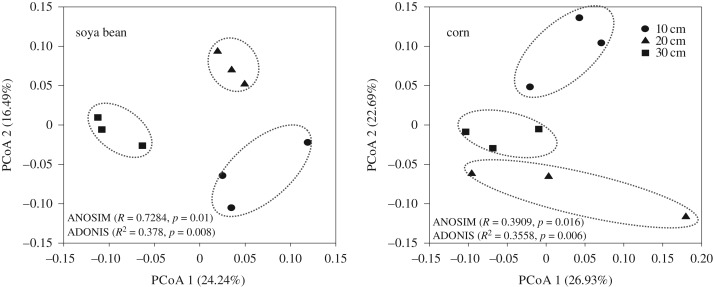
PCoA of soya bean and corn rhizosphere bacterial communities in different soil depths.

The variations in soya bean and corn rhizosphere bacterial communities caused by topsoil depths were investigated using PCoA based on Bray–Curtis distance. PCoA profiles yielded a separation among different topsoil depths both in soya bean and corn (figure 1). The PC1-axis and PC2-axis were 24.24% and 16.49%, and 26.93% and 22.69% for soya bean and corn, respectively. The results of ANOSIM and ADONIS analyses also showed significant community differences based on the OTUs derived from different topsoil depths both for soya bean and corn, indicating that compared with soil properties, soil bacterial communities were more sensitive to short-term reconstructed topsoil. This result further emphasized the importance of soil microbiological properties as early indicators of change in soil quality [28]. The community differences might be due to the different root growth characteristics induced by different topsoil depths, for example, topsoil depths could significantly increase soya bean root weight density and ratio of root length/root weight (Y Yang *et al.* 2019, unpublished), thus the significant changes detected in the soya bean and corn rhizosphere microbial communities in response to topsoil depth might be related to quantitative and qualitative changes in root biomass and rhizodeposition, which soil microbes depend on for food and energy. Whether and how these factors affect soil bacterial communities require further investigation.

### 3.4. Composition and specific bacterial taxa modulated by topsoil depths

**Figure 2.**
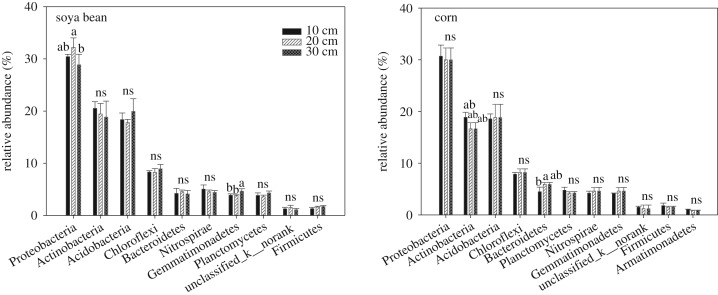
Significantly affected genera by topsoil depths in soya bean and corn.

A detailed distribution of the 10 most abundant bacterial phyla based on 16S rRNA sequence reads of all samples is shown in figure 2. The phyla Proteobacteria, Actinobacteria, Acidobacteria, Chloroflexi and Bacteroidetes occupied 79–83% and 80–82% of the bacterial sequences obtained from soya bean and corn, respectively. Proteobacteria was the most abundant phylum, accounting for 27–34% of the total reads in all the samples. Actinobacteria and Acidobacteria were the second and third dominant phyla, accounting for 15–20% and 15–23% of the total reads, respectively. In the case of soya bean, the distribution of Proteobacteria and Gemmatimonadetes was significantly different among different topsoil depths. While in the case of corn, the distribution of Actinobacteria and Bacteroidetes was significantly different among different topsoil depths. These four significantly modulated phyla in soya bean and corn among different topsoil depths were further analysed at the genus level by two-way ANOVA (table 3). In the case of soya bean, seven genera affiliated with Proteobacteria and two genera affiliated with Gemmatimonadetes were significantly affected by topsoil depths. While in the case of corn, three genera affiliated with Actinobacteria were significantly affected by topsoil depths. No genera from Bacteroidetes was significantly affected by topsoil depths. These different distributions of phyla and genera between soya bean and corn also supported the different responses of bacterial communities to topsoil thickness in soya bean and corn rhizosphere.

**Table 3.** Significantly changed genera in soya bean and corn among different topsoil depths.

						relative proportion of reads numbers (%)			ANOVA (*p*-values)
	phylum	class	order	family	genus	10 cm	20 cm	30 cm	soil depth
soya bean	Proteobacteria	Alphaproteobacteria	Rhizobiales	Bradyrhizobiaceae	*Bradyrhizobium*	1.78 ± 0.06	1.55 ± 0.09	1.62 ± 0.04	0.014
				Phyllobacteriaceae	*Mesorhizobium*	0.42 ± 0.06	0.49 ± 0.01	0.31 ± 0.07	0.016
				Xanthobacteraceae	norank_f__Xanthobacteraceae	0.65 ± 0.03	0.57 ± 0.02	0.55 ± 0.06	0.044
			Rhodospirillales	DA111	norank_f__DA111	0.47 ± 0.07	0.54 ± 0.03	0.68 ± 0.03	0.004
		Gammaproteobacteria	Xanthomonadales	Xanthomonadaceae	*Rhodanobacter*	1.45 ± 0.39	2.72 ± 0.50	0.71 ± 0.29	0.002
			Xanthomonadales	Xanthomonadaceae	unclassified_f__Xanthomonadaceae	0.28 ± 0.05	0.48 ± 0.11	0.36 ± 0.05	0.048
		Deltaproteobacteria	Myxococcales	Haliangiaceae	*Haliangium*	0.42 ± 0.04	0.61 ± 0.01	0.49 ± 0.06	0.005
	Gemmatimonadetes	Gemmatimonadetes	Gemmatimonadales	Gemmatimonadaceae	unclassified_f__Gemmatimonadaceae	0.20 ± 0.02	0.22 ± 0.05	0.32 ± 0.06	0.038
					*Gemmatimonas*	0.84 ± 0.16	0.83 ± 0.14	1.20 ± 0.16	0.042
corn	Actinobacteria	Actinobacteria	Gaiellales	norank_o__Gaiellales	norank_o__Gaiellales	3.57 ± 0.08	2.57 ± 0.39	3.11 ± 0.18	0.021
			Propionibacteriales	Nocardioidaceae	*Marmoricola*	0.44 ± 0.10	0.38 ± 0.04	0.64 ± 0.10	0.024
			Pseudonocardiales	Pseudonocardiaceae	*Pseudonocardia*	0.15 ± 0.02	0.16 ± 0.03	0.25 ± 0.06	0.048

Among the seven significantly modulated genera in soya bean, *Bradyrhizobium* constituting an important group of rhizobia, has served as a model system for studying host–microbe symbiotic interactions and N fixation due to its importance in agricultural productivity and global N cycling [29,30]. They are supposed to play crucial roles in degrading herbicides [31], promoting plant growth and attenuating the toxic effects of nickel and zinc [32]. Many strains have been isolated from soil and are available for use in commercial inoculants to increase soil quality and fertility [33]. *Rhodanobacter* was first proposed by Nalin *et al*. [34], and members of this genus have been isolated from forest soil [35], ginseng rhizosphere [36] and corn rhizosphere [37]. Some species have been shown to be acid tolerant, exhibiting complete denitrification [35,38]. Our previous study also showed that this genus was identified as denitrification bacteria in Mollisol [39], implying that it might contribute to N cycling in Mollisol and might be sensitive to environmental changes. Among the three significantly changed genera in corn, genera from the order Gaiellales had the most abundance. This order was recently identified with limited isolates available [40,41]. Members of Gaiellales can be reportedly found in various environments, such as thermal spring [42], soil [43] and the rhizosphere of rice [44]. This order contains aerobic microbes and those that can interact with complex polysaccharides derived from the plant [45]. Why erosion impacts the genera and possibly impacts the above bacterial function in relation to plant response to erosion needs to be clarified in the future studies.

### 3.5. Correlations between selected soil properties and significantly changed genus

Mantel test analyses revealed that the corn rhizosphere bacterial community was significantly correlated with soil $NO_{3}^{-}-N$ (*r* = 0.4089, *p* < 0.05), while the soya bean rhizosphere bacterial community was not significantly correlated with any soil biogeochemical characteristics. The correlations between selected soil properties and significantly changed genera are shown in electronic supplementary material, table S2. In the case of soya bean, among the seven significantly affected genera by topsoil depths, *Bradyrhizobium* was strongly correlated with microbial biomass nitrogen (MBN), available P and dissolved organic nitrogen (DON); *Haliangium* was strongly correlated with MBN; norank_f__Xanthobacteraceae was closely related with total C and unclassified_f__Xanthomonadaceae was closely related with MBN and DON. While in the case of corn, only one genus (norank_o__Gaiellales) was significantly correlated with pH. Thus, partial biogeochemical characteristic changes under different soil depth removal contribute to the change in the bacterial community by influencing specific genera.

## 4. Conclusion

This research confirmed that soil bacteria were more sensitive to topsoil removal conditions than to soil biogeochemical characteristics. The topsoil depths did not significantly influence the number of OTUs and α-diversity of bacterial communities in soya bean and corn, but influenced the 16S rRNA gene sequence copy numbers and soil respiration, especially in the case of corn. PCoA, ANOSIM and ADONIS analyses revealed significant community differences based on the OTUs derived from different topsoil depths both for soya bean and corn. Proteobacteria and Gemmatimonadetes, Actinobacteria and Bacteroidetes were significantly different among different topsoil depths for soya bean and corn, respectively. Further analysis showed that seven genera affiliated with Proteobacteria and two genera affiliated with Gemmatimonadetes were significantly affected by topsoil depths in soya bean, while in the case of corn, three genera affiliated with Actinobacteria were significantly affected by topsoil depths. These significant changes in the genera induced by topsoil depths may play a vital role in the agricultural productivity and global nutrient cycling of Mollisol. These bacterial function in relation to plant response to erosion needs to be clarified in the future studies. The comparison involving the undisturbed soil as a baseline also warrants further investigation.

## Supplementary Material

Table S1:

## Supplementary Material

Table S1:

## Data accessibility

The DNA sequences obtained in this study have been deposited into the GenBank short-read archive SRP151783.

## References

[1] Montgomery DR 2007 Dirt: the erosion of civilizations. Berkeley, CA: University of California Press.

[2] Lal R 1998 Soil erosion impact on agronomic productivity and environment quality. Crit. Rev. Plant. Sci. 17, 319–464. (10.1080/07352689891304249)

[3] Cotching WE, Hawkins K, Sparrow LA, McCorkell BE, Rowley W 2002 Crop yields and soil properties on eroded slopes of red ferrosols in North-west Tasmania. Soil Res. 40, 625–642. (10.1071/SR01062)

[4] Rosa D, Moreno JA, Mayol F, Bonsón T 2000 Assessment of soil erosion vulnerability in Western Europe and potential impact on crop productivity due to loss of soil depth using the ImpelERO model. Agr. Ecosyst. Environ. 81, 179–190. (10.1016/S0167-8809(00)00161-4)

[5] Yan BX, Tang J 2005 Study on black soil erosion rate and the transformation of soil quality influenced by erosion. Geogr. Res. 24, 499–506. (in Chinese).

[6] Hermans SM, Buckley HL, Case BS, Curran-Cournane F, Taylor M, Lear G 2017 Bacteria as emerging indicators of soil condition. Appl. Environ. Microbiol. 83, e02826-16. 27793827 10.1128/AEM.02826-16 PMC5165110

[7] Paul EA, Clark FE 1989 Soil microbiology and biochemistry. San Diego, CA: Academic Press Inc.

[8] Prosser J et al. 2007 The role of ecological theory in microbial ecology. Nat. Rev. Microbiol. 5, 384–392. (10.1038/nrmicro1643) 17435792

[9] Stroud JL, Paton GI, Semple KT 2009 Microbe–aliphatic hydrocarbon interactions in soil: implications for biodegradation and bioremediation. J. Appl. Microbiol. 102, 1239–1253. (10.1111/j.1365-2672.2007.03401.x) 17448159

[10] Li ZW, Xiao HB, Tang ZH, Huang JQ, Nie XD, Huang B, Ma WM, Lu YM, Zeng GM 2015 Microbial responses to erosion-induced soil physico-chemical property changes in the hilly red soil region of southern China. Eur. J. Soil Biol. 77, 37–44.

[11] Zhou KQ, Sui YY, Liu XB, Zhang XY, Jin J, Wang GH, Herbert SJ 2015 Crop rotation with nine-year continuous cattle manure addition restores farmland productivity of artificially eroded Mollisols in Northeast China. Field Crops Res. 171, 138–145. (10.1016/j.fcr.2014.10.017)

[12] Sui YY, Liu XB, Jin J, Zhang SL, Zhang XY, Herbert SJ, Ding GW 2009 Differentiating the early impacts of topsoil removal and soil amendments on crop performance/productivity of corn and soybean in eroded farmland of Chinese Mollisols. Field Crops Res. 111, 276–283. (10.1016/j.fcr.2009.01.005)

[13] Nazih N, Finlay-Moore O, Hartel GP, Fuhrmann J 2001 Whole soil fatty acid methyl ester (FAME) profiles of early soybean rhizosphere as affected by temperature and matric water potential. Soil Biol. Biochem. 33, 693–696. (10.1016/S0038-0717(00)00197-8)

[14] Vance ED, Brookes PC, Jenkinson DS 1987 An extraction method for measuring soil microbial biomass C. Soil Biol. Biochem. 19, 703–707. (10.1016/0038-0717(87)90052-6)

[15] Olsen S, Cole C, Watanabe F, Dean L 1954 Estimation of available phosphorus in soils by extraction with sodium bicarbonate. USDA Circular Nr 939. Washington, DC: US Gov. Print. Office.

[16] Lu RK 2000 Agricultural chemical analysis of the soil. Beijing, China: China Agricultural Science and Technology Press.

[17] Blagodatskaya E, Yuyukina T, Blagodatsky S, Kuzyakov Y 2011 Turnover of soil organic matter and of microbial biomass under C3–C4 vegetation change: consideration of ^13^C fractionation and preferential substrate utilization. Soil Biol. Biochem. 43, 159–166. (10.1016/j.soilbio.2010.09.028)

[18] Muyzer G, Waal EC, Uitterlinden AG 1993 Profiling of complex microbial populations by denaturing gradient gel electrophoresis analysis of polymerase chain reaction-amplified genes coding for 16S rRNA. Appl. Environ. Microbiol. 59, 695–700. 7683183 10.1128/aem.59.3.695-700.1993 PMC202176

[19] Piterina AV, Bartlett J, Pembroke JT 2010 Molecular analysis of bacterial community DNA in sludge undergoing autothermal thermophilic aerobic digestion (ATAD): pitfalls and improved methodology to enhance diversity recovery. Diversity 2, 505–526. (10.3390/d2040505)

[20] Liu JJ, Yu ZH, Yao Q, Hu XJ, Zhang W, Mi G, Chen XL, Wang GH 2017 Distinct soil bacterial communities in response to the cropping system in a Mollisol of northeast China. Appl. Soil. Ecol. 119, 407–416. (10.1016/j.apsoil.2017.07.013)

[21] Biddle JF, Fitz-Gibbon S, Schuster SC, Brenchley JE, House CH 2008 Metagenomic signatures of the Peru Margin subseafloor biosphere show a genetically distinct environment. Proc. Natl Acad. Sci. USA 105, 10583–10588. (10.1073/pnas.0709942105) 18650394 PMC2492506

[22] Xiong JB et al. 2012 Geographic distance and pH drive bacterial distribution in alkaline lake sediments across Tibetan Plateau. Environ. Microbiol. 14, 2457–2466. (10.1111/j.1462-2920.2012.02799.x) 22676420 PMC3477592

[23] Caporaso JG, Kuczynski J, Stombaugh J, Bittinger K, Bushman FD, Costello EK, Fierer N, Peña AG 2010 QIIME allows analysis of high-throughput community sequencing data. Nat. Methods 7, 335–336. (10.1038/nmeth.f.303) 20383131 PMC3156573

[24] Clarke KR 1993 Non-parametric multivariate analyses of changes in community structure. Aust. J. Ecol. 18, 117–143. (10.1111/j.1442-9993.1993.tb00438.x)

[25] Anderson MJ 2001 A new method for non-parametric multivariate analysis of variance. Austral. Ecol. 26, 32–46.

[26] Sui YY, Jin J, Liu XB, Zhang XY, Li YS, Zhou KQ, Wang GH, Di GL, Herbert SJ 2017 Soil carbon sequestration and crop yield in response to application of chemical fertilizer combined with cattle manure to an artificially eroded Phaeozem. Arch. Agron. Soil Sci. 63, 1510–1522. (10.1080/03650340.2017.1292032)

[27] Zhang JB, Song CC, Yang WY 2006 Land use effects on the distribution of labile organic carbon fractions through soil profiles. Soil Sci. Soc. Am. J. 70, 660–667. (10.2136/sssaj2005.0007)

[28] Bending GD, Putland C, Rayns F 2000 Changes in microbial community metabolism and labile organic matter fractions as early indicators of the impact of management on soil biological quality. Biol. Fertil. Soils 31, 78–84. (10.1007/s003740050627)

[29] Delamuta JRM, Ribeiro RA, Ormeño-Orrillo E, Melo IS, Martínez-Romero E, Hungria M 2013 Polyphasic evidence supporting the reclassification of *Bradyrhizobium japonicum* group Ia strains as *Bradyrhizobium diazoefficiens* sp. nov. Int. J. Syst. Evol. Microbiol. 63, 3342–3351. (10.1099/ijs.0.049130-0) 23504968

[30] VanInsberghe D, Maas RK, Cardenas E, Strachan RC, Hallam S, Mohn WW 2015 Non-symbiotic *Bradyrhizobium* ecotypes dominate North American forest soils. ISME J. 9, 2435–2441. (10.1038/ismej.2015.54) 25909973 PMC4611507

[31] Romdhane S, Devers-Lamrani M, Martin-Laurent F, Calvayrac C, Rocaboy-Faquet E, Riboul D, Cooper JF, Barthelmebs L 2016 Isolation and characterization of *Bradyrhizobium* sp. SR1 degrading two β-triketone herbicides. Environ. Sci. Pollut. Res. 23, 4138–4148. (10.1007/s11356-015-4544-1) 25903192

[32] Wani PA, Khan MS, Zaidi A 2007 Effect of metal tolerant plant growth promoting *Bradyrhizobium* sp. (vigna) on growth, symbiosis, seed yield and metal uptake by greengram plants. Chemosphere 70, 36–45. (10.1016/j.chemosphere.2007.07.028) 17723236

[33] Delamuta JRM, Ribeiro R, Ormeño-Orrillo E, Parma M, Melo I, Martínez-Romero E, Hungria M 2015 *Bradyrhizobium tropiciagri* sp. nov. and *Bradyrhizobium embrapense* sp. nov., nitrogen-fixing symbionts of tropical forage legumes. Int. J. Syst. Evol. Microbiol. 65, 4424–4433. (10.1099/ijsem.0.000592) 26362866

[34] Nalin R, Simonet P, Vogel TM, Normand P 1999 *Rhodanobacter lindaniclasticus* gen. nov., sp. nov., a lindane-degrading bacterium. Int. J. Syst. Evol. Microbiol. 49, 19–23. 10.1099/00207713-49-1-19 10028243

[35] Dahal RH, Kim J 2017 *Rhodanobacter humi* sp. nov., a novel acid tolerant and alkali tolerant gammaproteobacterium isolated from Kyonggi University forest soil. Int. J. Syst. Evol. Microbiol. 67, 1185–1190. (10.1099/ijsem.0.002452) 28073405

[36] Han SI, Lee YR, Kim JO, Whang KS 2016 *Terrimonas rhizosphaerae* sp. nov., isolated from ginseng rhizosphere soil. Int. J. Syst. Evol. Microbiol. 67, 391–395. 10.1099/ijsem.0.001639 27902263

[37] Wen X, Wang M, Ti J, Wu Y, Chen F 2017 Bacterial community composition in the rhizosphere of maize cultivars widely grown in different decades. Biol. Fertil. Soils 53, 221–229. (10.1007/s00374-016-1169-6)

[38] Prakash O, Green SJ, Jasrotia P, Overholt WA, Canion A, Watson DB, Brooks SC, Kostka JE 2012 *Rhodanobacter denitrificans* sp. nov., isolated from nitrate-rich zones of a contaminated aquifer. Int. J. Syst. Evol. Microbiol. 62, 2457–2462. (10.1099/ijs.0.035840-0) 22140175

[39] Yu ZH, Liu JJ, Li YS, Jin J, Liu XB, Wang GH 2018 Impact of land use, fertilization and seasonal variation on the abundance and diversity of *nirS*-type denitrifying bacterial communities in a Mollisol in Northeast China. Eur. J. Soil Biol. 85, 4–11. (10.1016/j.ejsobi.2017.12.001)

[40] Albuquerque L, França L, Rainey FA, Schumann P, Nobre MF, da Costa MS 2011 *Gaiella occulta* gen. nov., sp. nov., a novel representative of a deep branching phylogenetic lineage within the class Actinobacteria and proposal of *Gaiellaceae* fam. nov. and *Gaiellales* ord. nov. Syst. Appl. Microbiol. 34, 595–599. 21899973 10.1016/j.syapm.2011.07.001

[41] Ma B, Wang HZ, Dsouza M, Lou J, He Y, Dai ZM, Brookes PC, Xu JM, Gilbert JA 2016 Geographic patterns of co-occurrence network topological features for soil microbiota at continental scale in eastern China. ISME J. 10, 1891–1901. (10.1038/ismej.2015.261) 26771927 PMC5029158

[42] Rozanov AS et al. 2014 Molecular analysis of the benthos microbial community in Zavarzin thermal spring (Uzon Caldera, Kamchatka, Russia). BMC Genomics 15, S12 (10.1186/1471-2164-15-S12-S12) PMC4303939 25563397

[43] Kim YE et al. 2014 Metagenomic analysis of bacterial communities on Dokdo Island. J. Gen. Appl. Microbiol. 60, 65–74. (10.2323/jgam.60.65) 24859864

[44] Zecchin S, Corsini A, Martin M, Cavalca L 2017 Influence of water management on the active root-associated microbiota involved in arsenic, iron, and sulfur cycles in rice paddies. Appl. Microbiol. Biotechnol. 101, 6725–6738. (10.1007/s00253-017-8382-6) 28660288

[45] Szoboszlay M, White-Monsant A, Moe LA 2016 The effect of root exudate 7, 4′-dihydroxyflavone and naringenin on soil bacterial community structure. PLoS ONE 11, e0146555 (10.1371/journal.pone.0146555) 26752410 PMC4709137

